# Atmospheric nitrogen dioxide suppresses the activity of phytochrome interacting factor 4 to suppress hypocotyl elongation

**DOI:** 10.1007/s00425-024-04468-1

**Published:** 2024-07-03

**Authors:** Misa Takahashi, Atsushi Sakamoto, Hiromichi Morikawa

**Affiliations:** 1 Graduate School of Integrated Sciences for Life, Hiroshima University, Higashi, Hiroshima 739-8526 Japan; 2 School of Science, Hiroshima University, Higashi, Hiroshima 739-8526 Japan

**Keywords:** Air pollutant, Arabidopsis, Auxin pathway, Chromatin immunoprecipitation (ChIP) assay, Environment responses, Hypocotyl elongation, Nitrogen dioxide (NO_2_), Phytochrome-interacting factor 4 (PIF4)

## Abstract

**Main conclusion:**

Ambient concentrations of atmospheric nitrogen dioxide (NO_2_) inhibit the binding of PIF4 to promoter regions of auxin pathway genes to suppress hypocotyl elongation in Arabidopsis.

**Abstract:**

Ambient concentrations (10–50 ppb) of atmospheric nitrogen dioxide (NO_2_) positively regulate plant growth to the extent that organ size and shoot biomass can nearly double in various species, including *Arabidopsis thaliana* (Arabidopsis). However, the precise molecular mechanism underlying NO_2_-mediated processes in plants, and the involvement of specific molecules in these processes, remain unknown. We measured hypocotyl elongation and the transcript levels of *PIF4*, encoding a bHLH transcription factor, and its target genes in wild-type (WT) and various *pif* mutants grown in the presence or absence of 50 ppb NO_2_. Chromatin immunoprecipitation assays were performed to quantify binding of PIF4 to the promoter regions of its target genes. NO_2_ suppressed hypocotyl elongation in WT plants, but not in the *pifq* or *pif4* mutants. NO_2_ suppressed the expression of target genes of PIF4, but did not affect the transcript level of the *PIF4* gene itself or the level of PIF4 protein. NO_2_ inhibited the binding of PIF4 to the promoter regions of two of its target genes, *SAUR46* and *SAUR67*. In conclusion, NO_2_ inhibits the binding of PIF4 to the promoter regions of genes involved in the auxin pathway to suppress hypocotyl elongation in Arabidopsis. Consequently, PIF4 emerges as a pivotal participant in this regulatory process. This study has further clarified the intricate regulatory mechanisms governing plant responses to environmental pollutants, thereby advancing our understanding of how plants adapt to changing atmospheric conditions.

**Supplementary Information:**

The online version contains supplementary material available at 10.1007/s00425-024-04468-1.

## Introduction

Nitrogen dioxide (NO_2_) is often considered as a toxic gaseous air pollutant (Wellburn 1990). Upon combustion of fuels, nitrogen (N) in the air is oxidized into nitric oxide (NO), which is then rapidly converted into NO_2_ (Wellburn 1990; Wallington and Nielsen 1999). Atmospheric NO_2_ can be either detrimental or beneficial to plants depending on the concentration and plant species (Capron and Mansfield 1977; Sandhu and Gupta 1989; Wellburn 1990; Saxe 1994).

We recently reported that atmospheric NO_2_ at ambient concentrations (10–50 ppb) positively regulates Arabidopsis plant growth to increase shoot biomass (Takahashi et al. 2005, 2014a) and organ size (Takahashi et al. 2014a), and accelerates flowering (Takahashi et al. 2014b). We found that NO_2_ increased the leaf size and shoot biomass of Arabidopsis by 2.5-fold (Takahashi et al. 2014a, 2014b), and these increases were attributable to stimulation of both cell proliferation and cell enlargement by NO_2_ (Takahashi et al. 2014a). Nitrogen analyses of gaseous ^15^NO_2_-fed Arabidopsis plants have indicated that the contribution of NO_2_ to total plant nitrogen is minor (> 5%) and that NO_2_ may function as a signal rather than a nutrient (Takahashi et al. 2014a). However, the molecular mechanism underlying the responses of plant cells to NO_2_ is unknown.

It has been reported that the mechanism of hypocotyl elongation is distinct from the mechanism of biomass production (Ivakov et al. 2017; Costigliolo- Rojas et al. 2022). Consequently, investigations that focus on the effects of NO_2_ on hypocotyl elongation may not provide insights into the mechanisms underlying NO_2_-mediated biomass production. However, by utilizing a range of Arabidopsis mutants related to hypocotyl elongation, research investigating the key molecules involved in NO_2_-mediated processes can provide valuable information on how NO_2_ regulates plant cell behavior.

Phytochrome-interacting factor 4 (PIF4) is a basic helix–loop–helix (bHLH) transcription factor that functions as a central regulator in integrating environmental and developmental signals (Leivar and Quail 2011). It regulates the expression of various target genes involved in cell elongation and plant development. Mutants with a defective *PIF4* gene (*pif4*) exhibit a short hypocotyl, whereas *PIF*-overexpressing lines exhibit a long hypocotyl (Huq and Quail 2002). In the present study, we investigated the effects of NO_2_ on hypocotyl elongation of various Arabidopsis *pif* mutants to elucidate the involvement and functional significance of PIFs in mediating the physiological responses to NO_2_ exposure.

## Materials and methods

### Plant materials and growth conditions

As plant materials for the present study we used *Arabidopsis thaliana* (L.) Heynh. (accession Col-0) as the wild type (WT) and five mutants: *pif1-1* (designated as *pif1*) (Huq et al. 2004), *pif3* (*pif3*) (Monte et al. 2004), *pif5-3* (*pif5*) (Khanna et al. 2007), *pif4-2* (*pif4*), and the quadruple mutant *pif1pif3pif4pif5* (*pifq*) (Leivar et al. 2008). We also used the following PIF overexpressors: PIL5OX (*35S::PIF1:*MYC) (designated as *35S::PIF1*) (Oh et al. 2006), PIFF3:MYC (*35S::PIF3*) (Ni et al. 2013), *35S:PIF4*-MYC (*35S::PIF4*), PIFF5:HA (*35S::PIF5*) (Pfeiffer et al. 2014), and *PIF4p::PIF4*-HA/*pif4* (*PIF4::PIF4*-HA) (Yamashino et al. 2013). Seeds were surface-sterilized for 5 min with 1.0% sodium hypochlorite, rinsed in pure water (18.0 MΩ), imbibed, cold treated at 4 °C, and sown in a rectangular plastic tray (22 × 5 × 20 cm in width, height, and depth, respectively) containing vermiculite and perlite (1:1, v/v). Trays were transferred into a glass-walled NO_2_ exposure chamber (1.3 × 1.0 × 0.65 m in width, height, and depth, respectively; NOX-1000-SCII, Nippon Medical & Chemical Instruments Co., Osaka, Japan) and cultivated in a growth room according to Takahashi et al. (2014a). The temperature, CO_2_ concentration, and relative humidity in the chamber were set at 22 ± 0.1 °C, 360 ± 30 ppm, and 70% ± 1.5%, respectively. Air entering the chamber (at 1 L min^–1^) was stripped of NO, NO_2_, and O_3_ (to 0 ppb) using activated charcoal and NaMnO_4_ (PureliteE30; Nippon Puretec Co., Tokyo, Japan). Trays were irradiated with fluorescent light (70 µmol photons m^2^ s^–1^) in a 16-h light/8-h dark cycle, and seeds were allowed to germinate and grow for 2 days in air lacking NO_2_. Then, NO_2_ was added to air entering the exposure chamber at concentrations of 0 or 50 ± 0.3 ppb (designated as –NO_2_ control and + NO_2_-treated plants, respectively). Seedlings were irrigated twice weekly with Murashige and Skoog (MS) medium (Murashige and Skoog 1962) with half-strength inorganic salts and grown for up to another 26 days in the chamber.

### Analysis of hypocotyl length

We harvested seedlings of –NO_2_ control plants and + NO_2_-treated plants at 3–14 days of age; hypocotyl length was determined according to Fankhauser and Casal (2004). Seedlings were sandwiched between two acetate sheets (Holbein Works, Ltd., Osaka, Japan) and scanned using a flatbed scanner (CanoScan 5600F, Canon, Tokyo, Japan) at a resolution of 600 dpi. Hypocotyl length was analyzed using ImageJ software (National Institute of Health, Bethesda, MA, USA).

### Analysis of shoot biomass

Shoots harvested from 4-week-old plants were washed with pure water, lyophilized, and then weighed (Takahashi et al. 2005).

### RNA extraction and quantitative reverse-transcription polymerase chain reaction (qRT-PCR) analysis

Shoots of 8-day-old –NO_2_ control and + NO_2_-treated plant seedlings were harvested at ZT8, frozen in liquid N, and stored at –80 °C until use (where ZT (zeitgeber) is the number of hours from dawn). Frozen shoots were ground in liquid N using a mortar and pestle, and homogenized. Total RNA was then extracted using the NucleoSpin RNA Kit (Macherey–Nagel, Düren, Germany) following the manufacturer’s instructions. The total RNA (1 μg) was reverse-transcribed into cDNA with ReverTra Ace qPCR RT Master Mix (Toyobo Co., Ltd., Osaka, Japan) in a 10-µL reaction volume. qRT-PCR was performed using the Kapa SYBR FAST qRT-PCR Kit (Kapa Biosystems, Wilmington, MA, USA) and the CFX Connect Real-Time PCR Detection System (Bio-Rad Laboratories, Hercules, CA, USA) following the manufacturers’ instructions according to Takahashi et al. (2014a). Relative gene transcript levels were calculated using the comparative ΔΔ^–CT^ method (Livak and Schmittgen 2001); the gene encoding protein phosphatase 2A (*PP2A*) was used as the reference gene.

### Protein extraction and immunoblot analysis

Shoots from 9-day-old *PIF4*::*PIF4*-HA seedlings were harvested at ZT8, frozen in liquid *N*, and stored at – 80 °C until use. Frozen shoots were ground in liquid *N* using a mortar and pestle and homogenized with 0.1 mL extraction buffer (per 100 mg tissue) containing 50 mM Tris–HCl (pH 7.5), 150 mM NaCl, 10 mM MgCl_2_, 1 mM EDTA, 10 mM NaF, 2 mM Na_3_VO_4_, 25 mM glycerol phosphate, 10% glycerol, and 0.1% Nonidet P-40, containing 1 mM phenylmethylsulfonyl fluoride, 1 × cOmplete ULTRA protease inhibitor cocktail (Roche, Mannheim, Germany), and 20 µM MG132. The homogenate was centrifuged twice at 16,000 g for 10 min at 4 °C, and the resulting supernatant was used for immunoblot analysis. The homogenate protein content was determined following the method of Bradford (1976), with bovine serum albumin as the standard.

Proteins (30 μg/lane) were separated by sodium dodecyl sulfate polyacrylamide gel electrophoresis (SDS–PAGE) in a 10% polyacrylamide gel according to the method of Laemmli (1970) and transferred onto polyvinylidene difluoride membranes (Immobilon-P, Millipore, Billerica, MA, USA) using an electroblotter (Atto, Tokyo, Japan) according to the manufacturer’s instructions. The proteins that reacted with a monoclonal antibody against Human influenza hemagglutinin (HA)-tag (M180-7, MBL, Tokyo, Japan) diluted to 1:1000 or against Actin-11 (AS10 702, Agrisera, Vännäs, Sweden) diluted to 1:2000 on the membrane were detected according to Takahashi et al. (2015).

### Chromatin immunoprecipitation (ChIP)–qPCR assay

The ChIP assays were conducted following the method of Kaufmann et al. (2010) with modifications. Briefly, shoots (0.5 g) harvested from 9-day-old Arabidopsis plants harboring *PIF4::PIF4*-HA or WT (control) plants at ZT8 were fixed in phosphate-buffered saline (PBS) containing 1% (v/v) formaldehyde by vacuum infiltration. Formaldehyde was quenched with 0.1 M glycine under vacuum. After grinding the plant material in liquid N and filtering using Miracloth (Millipore), nuclei were isolated, and isolated chromatin was sheared using a QSonica Q700 cup horn sonicator (QSonica, Newtown, CT, USA) at 70% amplification, with 10 cycles of 30 s sonication followed by 30 s cooling. After sonication, the extract was centrifuged at 21,880 g for 10 min at 4 °C. After chromatin isolation, the chromatin concentration was measured, and immunoprecipitation was performed using Dynabeads (Invitrogen, Carlsbad, CA, USA) coated with rat anti-HA (Roche, Basel, Switzerland). Cross-links were reversed by incubation at 65 °C for 12 h, and DNA was purified using the IPure V2 kit (Diagenode, Liège, Belgium) and eluted in 100 μL Tris–EDTA (pH 8.0). The ChIP–qPCR analyses were conducted using 1 μL eluted DNA solution and specific primers, according to the manufacturer’s instructions (Diagenode) (Table S1). The ChIP–qPCR data are presented as relative amounts of immunoprecipitated DNA compared to input or % input.

### Statistical analyses

GraphPad Prism 8.0 software (GraphPad Software, La Jolla, CA, USA) was used for all statistical analyses. Student’s *t*-test or Mann–Whitney *U* test was used to compare two groups. Data were subjected to one-way ANOVA followed by Tukey’s post hoc test, or two-way ANOVA (NO_2_ treatment, genotype, N × G interaction) followed by Tukey’s post hoc test. Differences at *P* < 0.05 were considered to be statistically significant. Significant differences among groups are indicated by letters in figures.

## Results and discussion

### NO_2_ suppresses hypocotyl elongation in WT but not in *pif4* mutants

**Fig. 1:**
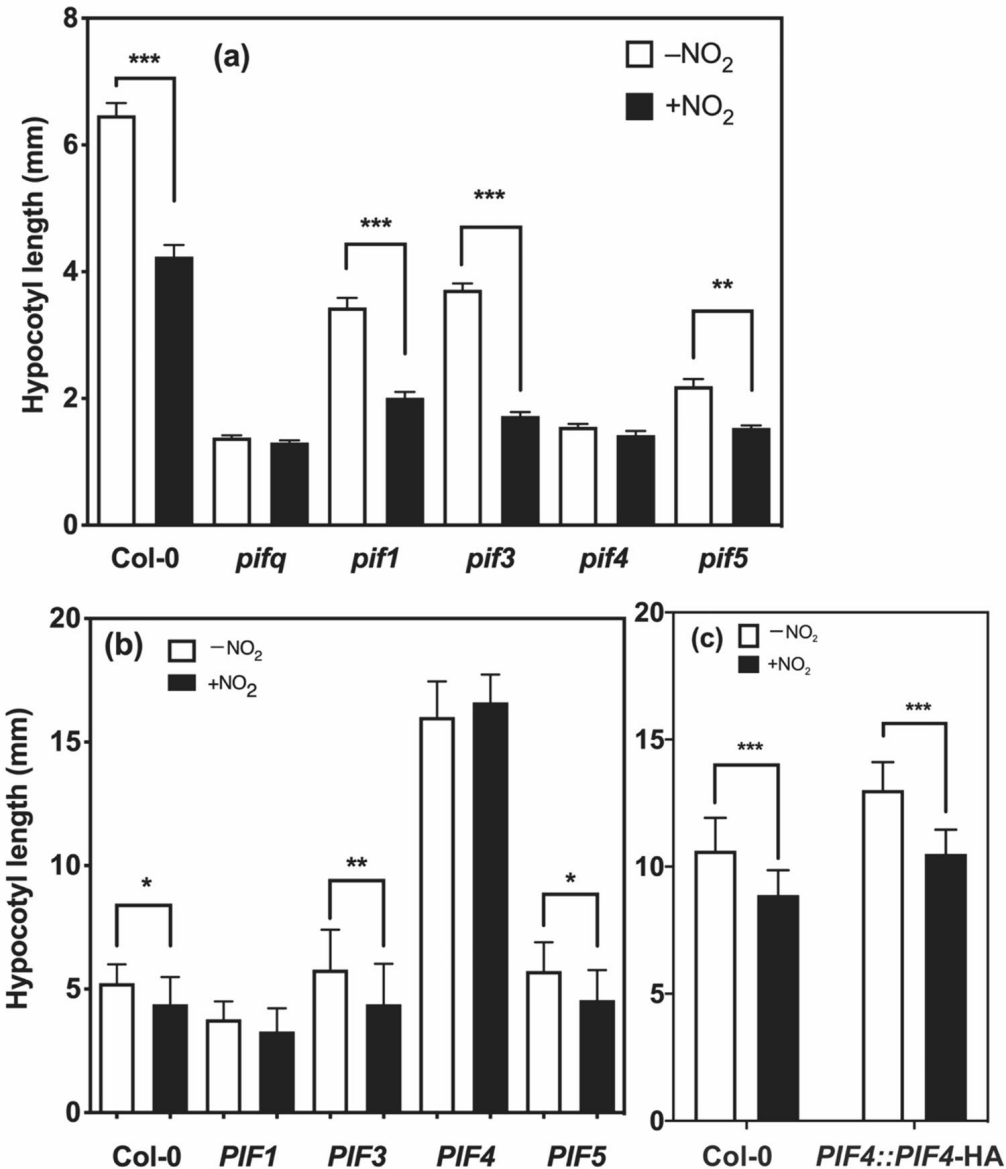
Hypocotyl elongation in the presence and absence of NO_2_ in wild-type (WT; Col-0) plants; mutants with defective *PIF* genes (single *pif* mutants *pif1*, *pif3*, *pif5*, and *pif4*, and the quadruple mutant *pifq*); and PIF overexpressors (*35S::PIF1*, *35S::PIF3*, *35S::PIF4*, *35S::PIF5*, and *PIF4::PIF4-*HA). After sowing, plants were grown in air without NO_2_ for the first 2 days and then grown for up to 12 additional days in air with 50 ± 0.3 ppb NO_2_ (+ NO_2_ plants) or without NO_2_ (0 ppb NO_2_) (–NO_2_ plants) under a 16-h light/8-h dark photoperiod. Hypocotyl lengths of WT and *pif* mutants (**a**) and overexpressors (**b** and **c**) are shown. Values are mean ± standard deviation (SD) of data from more than ten independent biological replicates. Statistical significance was assessed using one-way ANOVA. ^*^P < 0.05; ^**^P < 0.01; ^***^P < 0.001

*PIF4* encodes a bHLH transcription factor known as a phytochrome-interacting factor, which plays a central role in integrating environmental and developmental signals (Leivar and Quail 2011); it regulates the expression of various target genes involved in cell elongation and plant development. We investigated the effect of NO_2_ on the hypocotyl length of a *pif4* mutant with defective hypocotyl elongation (Huq and Quail 2002). First, we determined the effect of NO_2_ on the hypocotyl length of WT at 3–14 days after sowing (DAS). The hypocotyl length reached a plateau at 11–13 DAS in both + NO_2_ and –NO_2_ plants, and was 1.7-fold higher in –NO_2_ plants than in + NO_2_ plants (Fig. S1). Although NO_2_ inhibited hypocotyl elongation in WT, it had no significant effect on the hypocotyl length of the *pif4* mutant (Fig. 1). NO_2_ inhibited hypocotyl elongation in other single *pif* mutants, such as *pif1*, *pif3*, and *pif5*, but did not significantly inhibit hypocotyl elongation in the quadruple mutant *pifq*. These results indicate that *PIF4* uniquely eliminates NO_2_-triggered inhibition of hypocotyl elongation in Arabidopsis. This finding suggests that *PIF4* is involved in the mechanism underlying NO_2_-mediated physiological processes in Arabidopsis. The inhibition of hypocotyl elongation by NO_2_ was still observed in WT grown under higher light intensity (Fig. S2). This indicated that the effect of NO_2_ on hypocotyl elongation is not generally limited by the existing hypocotyl length. This suggests that the *pifq* and *pif4* mutants are indeed responsive to NO_2_.

We speculated that NO_2_ may suppress PIF4 activity transcriptionally and/or translationally to suppress Arabidopsis hypocotyl elongation. Notably, hypocotyl length was shortest in the *pif4* or *pifq* mutants among all the lines tested in this study.

The hypocotyl elongation of overexpressors in the presence and absence of NO_2_ was very similar to that of WT, except that the inhibitory effect of NO_2_ on hypocotyl elongation in *PIF1-* and *PIF4*-overexpressors was not statistically significant (Fig. 1b). The fact that the *PIF4*-overexpressors produced the longest hypocotyls among all the lines tested in this study in the presence or absence of NO_2_ is notable, given that *PIF4* uniquely regulates NO_2_-induced hypocotyl elongation among *PIF* genes, as mentioned above. PIF1 has a limited regulatory role in the modulation of hypocotyl elongation; instead, its predominant function lies in the suppression of seed germination (Oh et al. 2004; Leiver and Quail 2011). As shown in Fig. 1b, among all the *PIF*-overexpressing lines, the *PIF1* overexpressor produced the shortest hypocotyls in the presence or absence of NO_2_.

The hypocotyl length of one of the PIF4-overexpressors (*35S::PIF4-*Myc) was almost the same in the presence or absence of NO_2_. This may have been because of the substantial abundance of the PIF4 protein in the overexpressor, resulting in a diminished impact of NO_2_ on hypocotyl elongation. The hypocotyl of the *PIF4*-overexpressing line in which *PIF4* was under the control of its native promoter (*PIF4::PIF4:*HA) was shorter in the presence of NO_2_ than in the absence of NO_2_ (Fig. 1c).

### NO_2_ suppressed the expression of PIF4 target genes

**Fig. 2:**
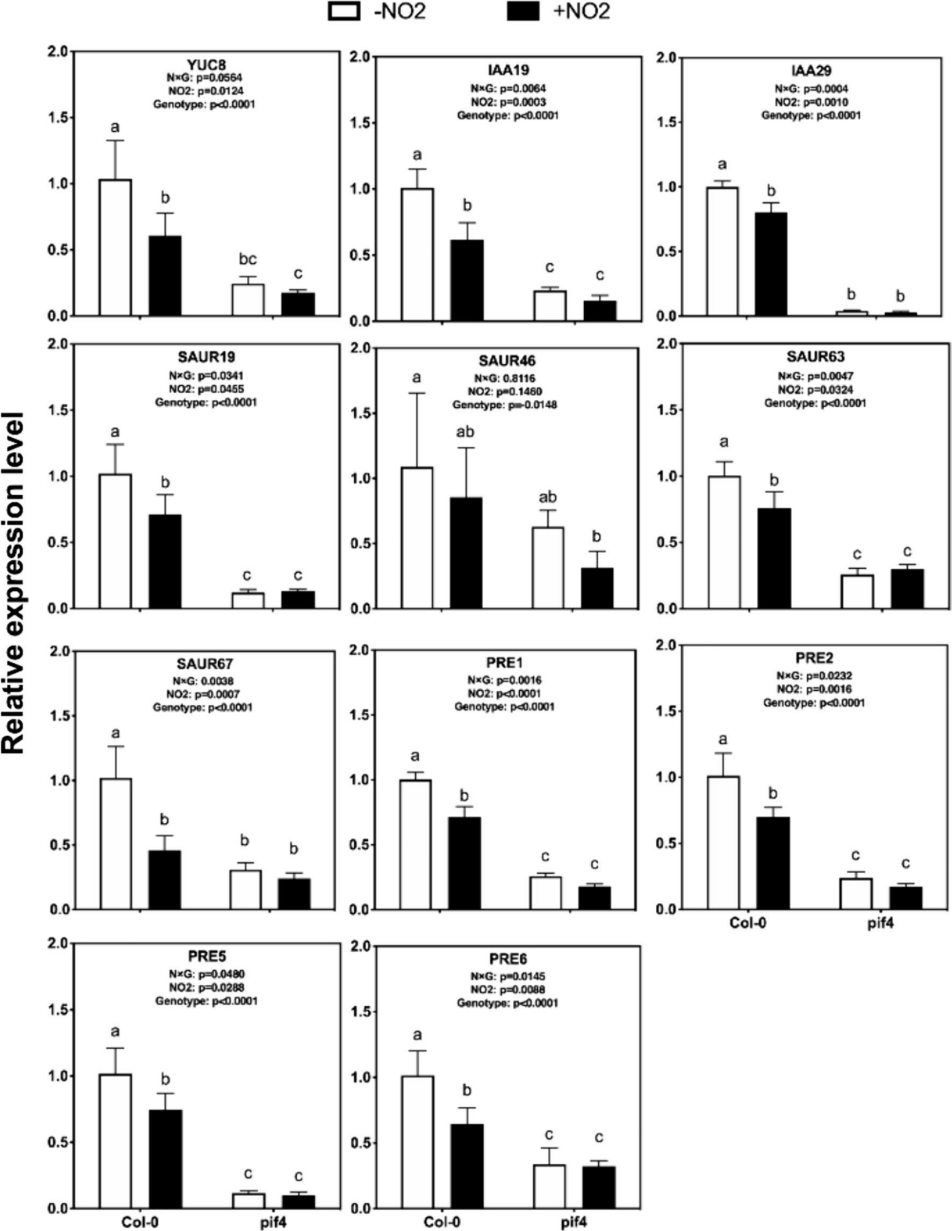
Transcript levels of 11 genes under regulation of PIF4 in wild-type control (Col-0) and *pif4* mutant plants grown in the absence (–NO_2_) and presence (+ NO_2_) of NO_2_ as described in the legend of Fig. 1. Transcript levels were normalized to that of *PP2A* and are displayed relative to that in –NO_2_ plants (set to 1) as the control. Each bar represents mean ± SD of data from four independent biological replicates. Two-way ANOVA was used to analyze the effects of NO_2_ treatment and genotype on gene transcript levels. Different letters indicate significant differences

We hypothesized that the suppression of hypocotyl elongation by NO_2_ may result from inhibition of the transcription of the target genes of PIF4. To test this hypothesis, we selected 11 genes reported to be target genes of PIF4, namely *YUCCA8 (YUC8)*, *IAA19*, *IAA29*, *SAUR19*, *SAUR46*, *SAUR63*, *SAUR67*, *PRE1*, *PRE2*, *PRE5*, *PRE6* (Oh et al. 2012), and tested the effect of NO_2_ on their transcript levels in WT Arabidopsis (Col-0) using qRT-PCR (Fig. 2). All of the tested genes were significantly down-regulated by NO_2_ (Fig. 2). Thus, NO_2_ inhibited the transcriptional expression of a variety of auxin pathway genes under the control of PIF4 to suppress hypocotyl elongation.

We also examined the transcript levels of these 11 genes in WT and the *pif4* mutant (Fig. 2). The transcript levels of these 11 genes were lower in the *pif4* mutant than in WT in the absence of NO_2_, and NO_2_ exposure did not significantly alter their transcriptional responses (Fig. 2). A two-way ANOVA revealed a significant interaction between genotype and NO_2_ treatment for almost all the genes.

In this study, NO_2_ suppressed the expression of *YUC8*, *IAA19*, *IAA29*, *SAUR19*, *SAUR46*, *SAUR63*, *SAUR67*, *PRE1*, *PRE2*, *PRE5*, and *PRE6* (Fig. 2). The NO_2_-induced down-regulation of auxin-responsive genes (*IAA19*, *IAA29*, *SAUR19*, *SAUR46*, *SAUR63*, *SAUR67*) may result in a reduction of cell elongation, leading to a shortened hypocotyl length. Some of these genes play critical roles in processes that affect cell elongation, such as promoting auxin transport (Chae et al. 2012) and stimulating membrane acidification (Spartz et al. 2014). PRE proteins are a class of HLH proteins that function as a secondary repressor (Buti et al. 2020) and the expression of their encoding genes is activated by auxin (Zheng et al. 2017). *YUC8* is involved in the auxin biosynthesis pathway and is regulated by PIF4 in high-temperature-induced hypocotyl elongation (Sun et al. 2012). These results indicated that NO_2_ requires a functional *PIF4* gene to affect the expression of genes involving the auxin biosynthesis.

### NO_2_ did not affect *PIF4* transcription or PIF4-HA protein accumulation

**Fig. 3:**
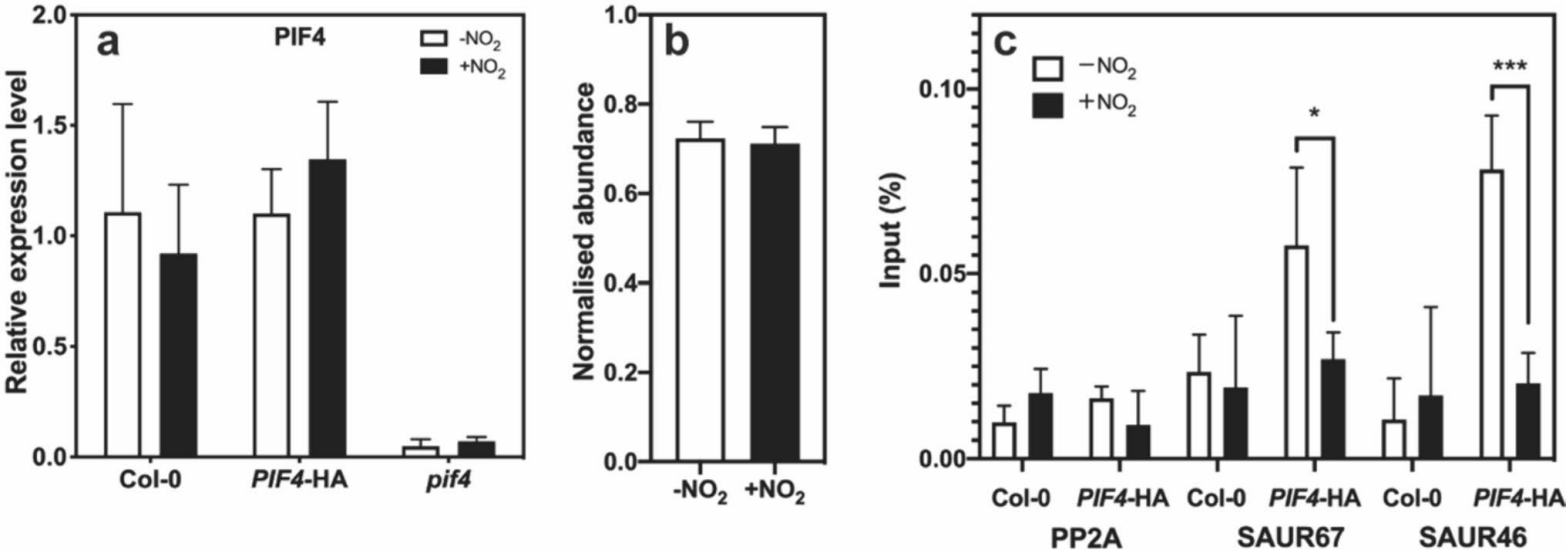
**a** Transcript levels of *PIF4* in Arabidopsis plants grown in the presence and absence of NO_2_. Plants were grown for 8 days as described for Fig. 1. Plants were harvested at ZT6 and total RNA extracted, followed by qPCR analysis to determine *PIF4* transcript levels. Transcript levels were normalized to the value in –NO_2_ WT plants (set to 1). Arabidopsis *PP2A* was used as an internal control. Each bar represents mean ± SD of data from four independent biological replicates. **b** Immunoblot analysis of PIF4-HA proteins in Arabidopsis *PIF4::PIF4*-HA plants. Plants were grown in the presence and absence of NO_2_ for 9 days as described for Fig. 1. Total proteins were extracted, and immunoblot analysis was performed using anti-HA-tag and anti-ACT11 (load control). Intensity of band around 35 kDa in the blot with anti-HA antibody (Fig. S4a) and intensity of band around 40 kDa in the blot with anti-actin antibody (Fig. S4c) were quantified. PIF4-HA protein amount was calculated from PIF4-HA signal normalized to actin (ACT11) signal. Each bar represents mean ± SD of data from four to five independent biological replicates. **c** Chromatin immunoprecipitation (ChIP) assays for *SAUR67* and *SAUR46* in *PIF4::PIF4*-HA Arabidopsis plants. Plants were grown in the presence and absence of NO_2_ for 9 days, as described for Fig. 1. Arabidopsis *PP2A* (load control) was used as an internal control. WT (Col-0) plants were used as a negative control. Plants were harvested at ZT6. Each bar represents mean ± SD of data from three independent technical replicates; *P < 0.05, ***P < 0.001 (two-way ANOVA)

Our results show that NO_2_ inhibited the transcriptional expression of auxin pathway genes. To determine whether this was attributable to down-regulation of *PIF4* transcription and/or PIF4 protein accumulation, we performed qRT-PCR and protein immunoblot analyses. The transcript levels of *PIF4* did not differ between –NO_2_ and + NO_2_ plants across the diurnal cycle (Fig. 3a, Fig. S3), demonstrating that the inhibition of the transcriptional expression of auxin pathway genes by NO_2_ is not due to down-regulation of *PIF4* transcript levels*.*

Since no anti-PIF4 antibody was available, we used a transgenic line expressing a fusion construct of PIF4 and the HA antigen (*PIF4::PIF4*-HA) (designated as PIF4-HA) (Yamashino et al. 2013). The amount of PIF4-HA was determined by immunoblot analysis using an anti-HA antibody according to Yamashino et al. (2013) (Fig. 3b, Fig. S4). The PIF4 protein levels did not differ between –NO_2_ and + NO_2_ plants. Together, these results indicate that NO_2_-induced down-regulation of *PIF4*-controlled auxin pathway genes was not because of a reduction in *PIF4* transcription or PIF4 protein accumulation.

### NO_2_ inhibited binding of PIF4 to the promoter of target genes

Despite the failure of NO_2_ to alter *PIF4* transcript or PIF4 protein levels, NO_2_ distinctly suppressed the expression of the target genes of PIF4. We, therefore, suspected that NO_2_ may alter the binding of PIF4 to the promoter regions of its target genes. To address this question, we performed ChIP assays using proteins extracted from *PIF4*-overexpressors grown in the presence and absence of NO_2_. The hypocotyl length of the *PIF4::PIF4*-HA transgenic line was shorter in the presence of NO_2_ than in the absence of NO_2_ (Fig. 1c), whereas the hypocotyl length elongation of *35S::PIF4-*Myc was not inhibited by NO_2_. Therefore, we used the *PIF4::PIF4*-HA transgenic line for ChIP analysis. Immunoprecipitation was performed using an anti-HA antibody.

The ChIP assays with the *SAUR46* and *SAUR67* genes clearly indicated distinct decreases in percent input values or relative amounts of immunoprecipitated DNA upon NO_2_ treatment (Fig. 3c). Overall, the ChIP assays of *PIF4::PIF4*-HA plant extracts using anti-HA antibody showed that NO_2_ decreased PIF4 binding to the promoter regions of target genes *SAUR67* and *SAUR46* by about one half. Both *SAUR46* and *SAUR67* are responsive to auxin (Nemhauser et al. 2006), suggesting that NO_2_ may affect gene expression through auxin pathways in Arabidopsis.

These findings indicate that NO_2_ may post-translationally modify the PIF4 protein to substantially inhibit its binding to the promoter regions of *SAUR67* and *SAUR46*.

To date, nitration of PIF4 proteins following exposure to NO_2_ has not been detected. Alternatively, given that the DELLA protein is a transcriptional co-repressor and DELLA blocks PIF4 transcriptional activity by binding to its DNA-recognition domain (de Lucas et al. 2008; Li et al. 2016), it is possible that NO_2_-induced down-regulation of PIF activity is mediated by increasing DELLA protein levels. Whether NO_2_ increases DELLA protein levels is currently under investigation.

In this study, the ChIP assay results showed that PIF4 binding to promoters of genes other than *SAUR67* and *SAUR46* was not affected by NO_2_ (Fig. S5). These findings suggest that, beyond PIF4, additional proteins like PIF4-interacting proteins such as brassinazole resistant 1 (BZR1) and auxin response factor 6 (ARF6) may play roles in the transcriptional regulation of the 11 genes examined in this study. NO_2_ may exert inhibitory effects on these proteins, leading to the suppression of gene expression.

### The *pif4* mutant did not exhibit a shoot biomass response to NO_2_ treatment

Given the involvement of PIF4 in the inhibition of hypocotyl elongation by NO_2_, we investigated the effects of NO_2_ on biomass in the *pif4* mutant to determine whether PIF4 is also involved in stimulation of shoot biomass by NO_2_. To consolidate this, we evaluated the impact of NO_2_ on shoot biomass in both WT and the *pif4* mutant.

**Fig. 4:**
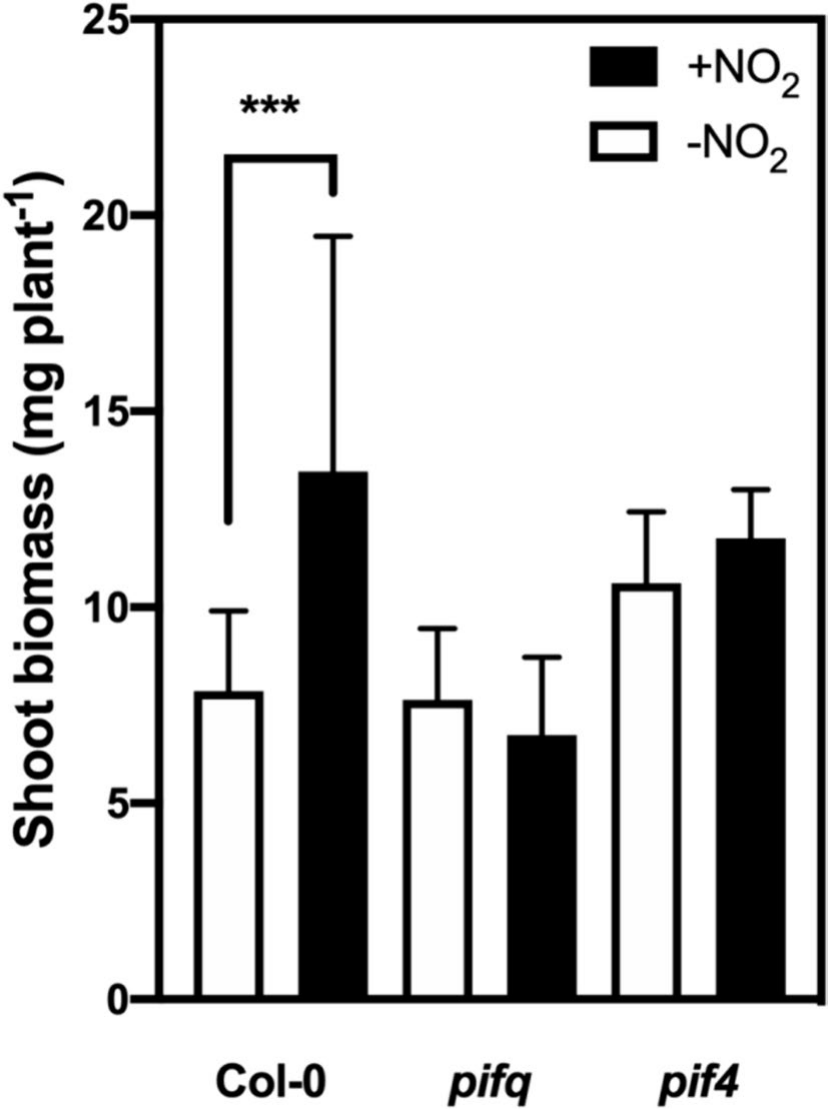
Shoot biomass of the wild-type control (WT; Col-0) as well as the *pif4* and *pifq* mutant plants grown in the presence and absence of NO_2_ as described in the legend of Fig. 1. For the 4-week-old WT, *pif4*, and *pifq* plants, the shoot biomass is presented as the mean ± SD of the data from 10 independent biological replicates. Statistical significance was assessed using a one-way ANOVA followed by Tukey’s post hoc test: ^***^P < 0.001

In the WT plants, NO_2_ notably increased shoot biomass at 28 days after sowing. In contrast, there were no significant differences in shoot biomass between –NO_2_ and + NO_2_ conditions in the *pif4* mutant (Fig. 4). The shoot biomass of the *pif4* mutant was almost the same as that of the WT in the absence of NO_2_. The results obtained using the *pifq* mutant were almost the same as those obtained using the *pif4* mutant. These results suggested that PIF4 is involved in the response to NO_2_ not only in the hypocotyl, but also in the shoot. The opposite effects of NO_2_ on the shoot and hypocotyl are indicative of organ-specific response mechanisms to NO_2_. Costigliolo-Rojas et al. (2022) recently reported the organ-specific regulatory mechanisms in the shoot and hypocotyl under shaded and warm conditions. Consistent with the results of earlier studies by Shimizu et al. (2016) and Kim et al. (2020) that demonstrated the tissue-specific function of PIF4, our findings suggest that PIF4 may modulate organ-specific regulatory pathways in response to NO_2_.

### NO_2_ inhibited hypocotyl elongation through PIF4 signaling

In this study, we investigated the effects of NO_2_ on hypocotyl elongation and found that NO_2_ suppressed hypocotyl elongation in Arabidopsis WT grown in the light (Fig. 1).

Significant suppression of hypocotyl elongation by NO_2_ was observed in WT and the *pif1*, *pif3*, and *pif5* mutants (Fig. 1a), but not in the *pifq* or *pif4* mutants (Fig. 1a). PIF1, PIF3, PIF4, and PIF5 have been reported to act redundantly in etiolated seedlings (Zhang et al. 2013). In green seedlings, PIF4 and PIF5 are involved in hypocotyl elongation (Hornitschek et al. 2012). However, such PIF redundancy is not involved in the effects of NO_2_ on hypocotyl elongation. PIF4 is similarly essential in responses to NO_2_ and high temperature (Koini et al. 2009).

PIF4 is a bHLH transcription factor involved in the integration of multiple signals for plant growth regulation. It is activated by various environmental factors including light and temperature, and hormonal signals; it regulates target genes involved in cell elongation (Koini et al. 2009). Recently, it has been revealed that PIF4 integrates cues of nitrate responses (Pereyra et al. 2023). Thus, whether alone or in combination with other proteins, PIF4 might regulate the expression of genes involved in auxin pathways in the response to NO_2_ exposure.

### NO_2_ inhibited binding of PIF4 to the promoter regions of target genes

Atmospheric NO_2_ did not affect hypocotyl length in the *pif4* mutant (Fig. 1a); in fact, *pif4* had the shortest hypocotyl length among all the lines tested in this study. Therefore, we presumed that NO_2_ suppresses *PIF4* gene expression or PIF4 protein activity to suppress hypocotyl elongation. However, qRT-PCR and immunoblot analyses showed that NO_2_ did not affect the *PIF4* transcript level or PIF4 protein level (Fig. 3a and b, respectively). However, NO_2_ reduced the ability of PIF4 to bind to the promoters of its target genes (Fig. 3c); therefore, the PIF4 protein may be modified post-translationally by NO_2_.

NO_2_ is a potent, non-discriminating nitrating agent as well as a hydrophobic molecule, and cell membranes are not significant barriers to NO_2_ transport (Signorelli et al. 2011). In in vivo studies, NO_2_ has been shown to be involved in protein tyrosine nitration (Kolbert et al. 2017) and protein nitrosylation (Heo and Campbell 2004). Protein tyrosine nitration is a covalent post-translational protein modification that plays a vital role in cell physiological processes including cellular signaling (Rubbo and Radi 2008; Ischiropoulos 2009). We recently identified nitratable proteins in Arabidopsis and showed that its proteins are selectively nitrated (Takahashi et al. 2015). Protein nitration has been shown to inhibit protein activity and interactions (Álvarez et al. 2011; Lozano-Juste et al. 2011). It can prevent proteins from exercising their normal functions such as phosphorylation or mimic the structural changes that occur after phosphorylation (Lindermayr and Durner 2009). For example, nitration of the β-subunit of F1-ATPase results in reduced ATPase activity (Fujisawa et al. 2009). Previously, we reported the nitration of PsbO1 by NO_2_ and showed that this resulted in inhibition of oxygen evolution in Arabidopsis leaves (Takahashi et al. 2017a,b). Nitrated proteins may also be involved in cellular signaling underlying NO_2_-regulated plant growth (Takahashi and Morikawa 2019).

NO_2_ is involved in the nitrosylation of plant proteins (Heo and Campbell 2004; Keszler et al. 2010). S-nitrosylation of proteins is a typical redox signaling mechanism (Sanz et al. 2015). Many proteins are nitrosylated in plants fumigated with NO_2_ (Heo and Campbell 2004; Kovacs and Lindermayr 2013; Takahashi et al. 2015). Therefore, nitrosylated proteins may play a role in cellular signaling underlying NO_2_-regulated plant growth (Takahashi et al. 2005, 2014a). NO-mediated up-regulation of auxin signaling through S-nitrosylation of the TIR1 auxin receptor promotes TIR1-Aux/IAA, facilitating Aux/IAA degradation and subsequently enhancing activation of gene expression (Terrile et al. 2012).

To integrate a variety of environmental, phytohormonal, and developmental signals, PIF4 cooperatively interacts with other protein factors such as ARF6 and BZR1 (positive regulatory factors) (Oh et al. 2014, 2016) and DELLA (a negative regulatory factor) (Li et al. 2016). NO_2_, which can permeate from the apoplast through the plasma membrane to reach the cytosol and nucleus, may modify these proteins, eventually suppressing binding of PIF4 to the promoters of its target genes. These modifications may inhibit the formation of complexes that include PIF4 and prevent PIF4 from binding to the promoter regions of its target genes. Determining the mechanism by which NO_2_ modifies these protein factors that play important roles in controlling PIF4 activity is an important and intriguing objective for future studies.

### Supplementary Information

Below is the link to the electronic supplementary material.

Supplementary file1 (DOCX 4670 KB)

## Data Availability

Data sharing not applicable to this article as no datasets were generated or analyzed during the current study.

## Abbreviations

ChIP: Chromatin immunoprecipitation
HA: Hemagglutinin
N: Nitrogen
NO_2_: Nitrogen dioxide
PIF: Phytochrome-interacting factor
PP2A: Protein phosphatase 2A
PRE: Paclobutrazol resistance
SAUR: Small auxin up-regulated
YUC8: YUCCA8
