# Supplementary material for: Atmospheric nitrogen dioxide suppresses the activity of phytochrome interacting factor 4 to suppress hypocotyl elongation
Source: Planta. 2024 Jul 3;260(2):42. doi: 10.1007/s00425-024-04468-1 (PMC11222245; doi:10.1007/s00425-024-04468-1)
Supplement: Supplementary file 1 — Supplementary file1 (DOCX 4670 KB) [file 425_2024_4468_MOESM1_ESM.docx]

**Article title:**

**Atmospheric nitrogen dioxide suppresses the activity of PHYTOCHROME INTERACTING FACTOR 4 to suppress hypocotyl elongation**

**Journal name: Planta**

**Author names: Misa Takahashi, Atsushi Sakamoto and Hiromichi Morikawa**

**Affiliation and e-mail address of the corresponding author:** Graduate School of Integrated Sciences for Life, Hiroshima University

**misat@hiroshima-u.ac.jp**


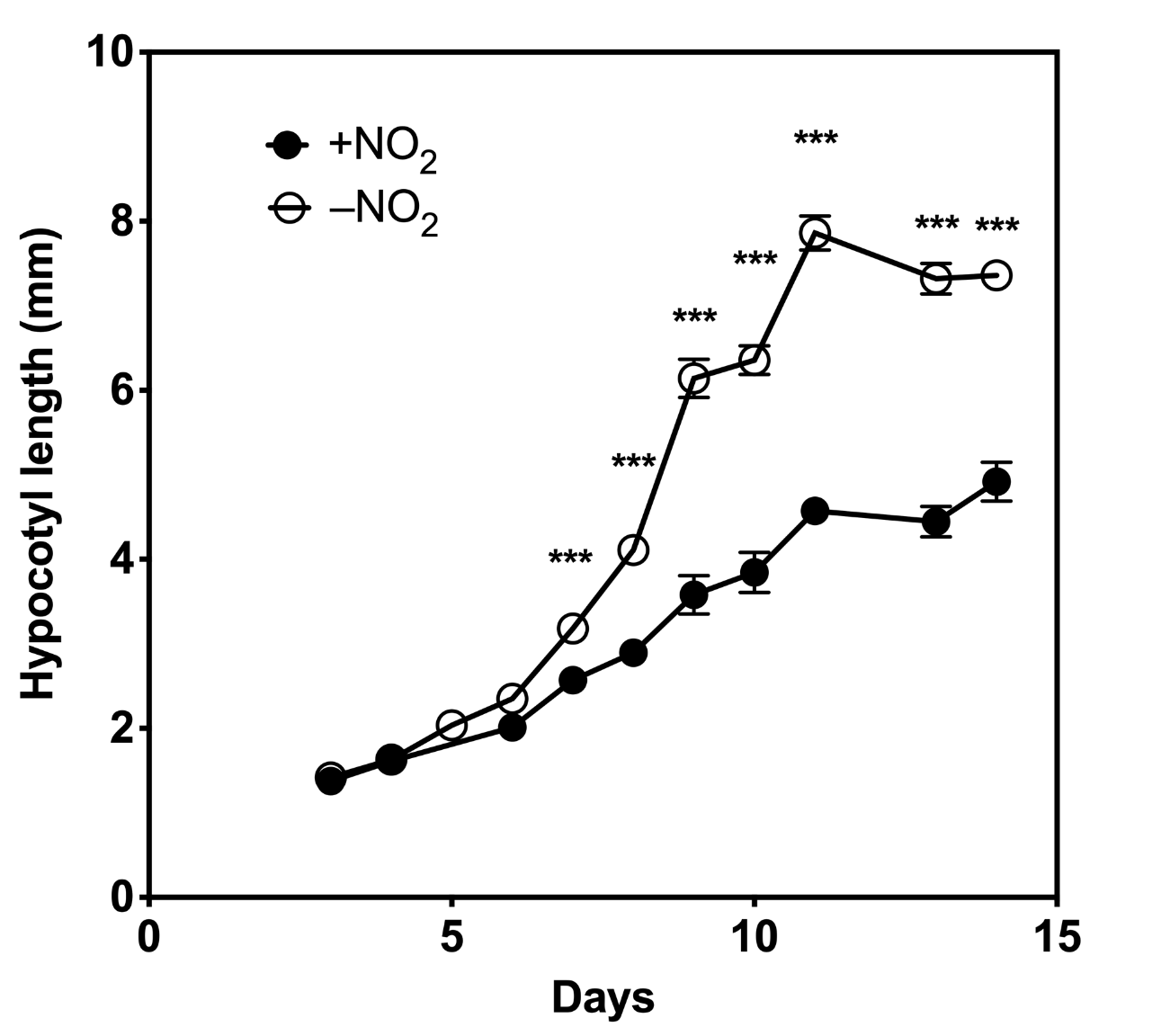


**Fig. S1.** Hypocotyl lengths of 3- to 14-day-old Arabidopsis plants. After sowing, plants were grown in air with (+NO_2_-treated plants, black) or without (–NO_2_ control plants, white) 50 ± 0.3 ppb NO_2_. Values are mean ± standard deviation (SD); *n* ≥ 15. Statistical significance was assessed using a Mann–Whitney U-test: ^*^*p* < 0.05; ^***^*p* < 0.001.


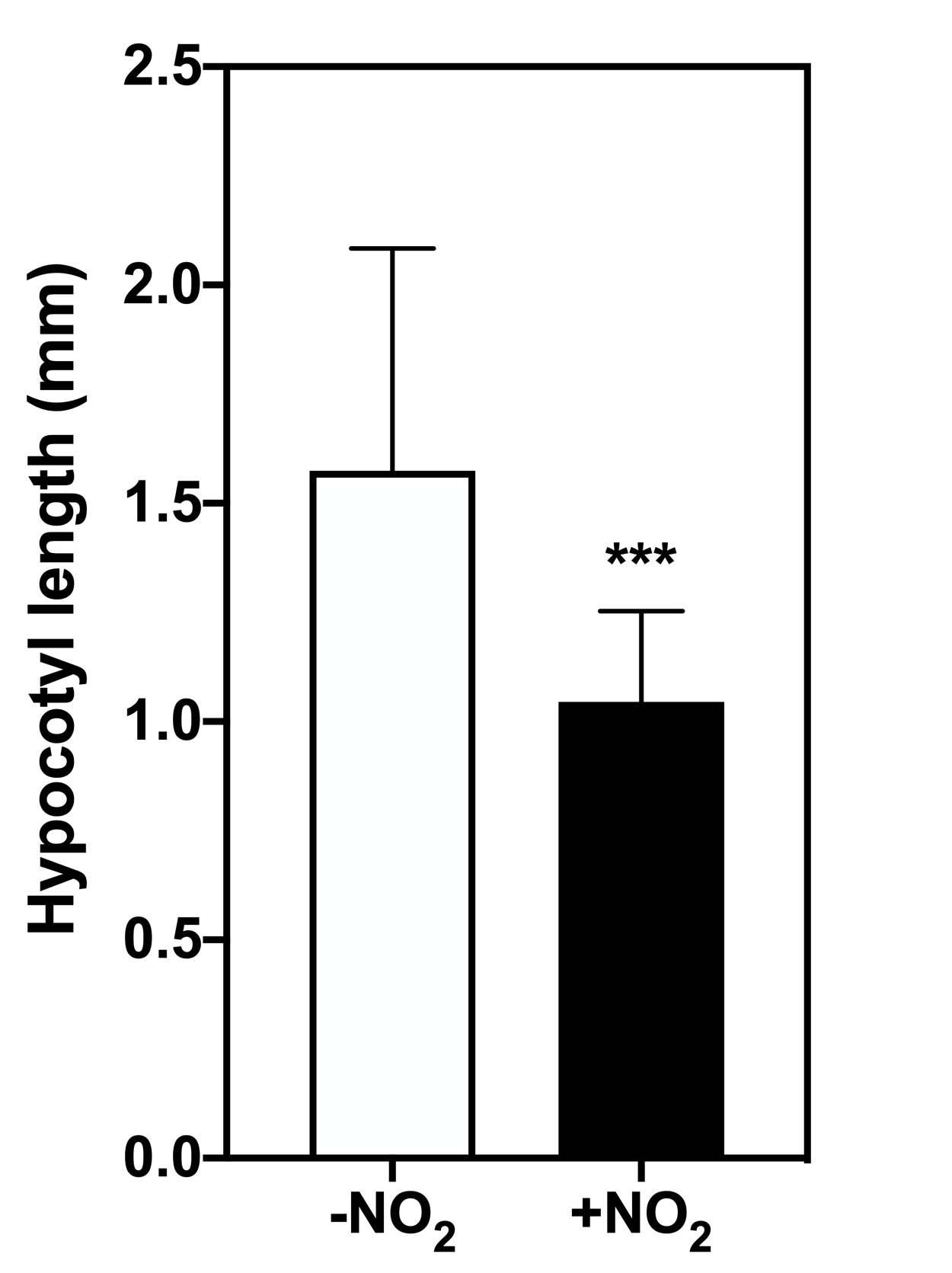


Fig. S2. NO_2_ suppressed hypocotyl elongation in WT grown under higher light intensity (250 µmol photons m^2^ s^–1^). Plants were grown in the presence and absence of NO_2_ for 12 days as described in Fig. 1. Values are mean ± standard deviation (SD) of data from more than ten independent biological replicates. Statistical significance was assessed using Student’s *t*-test. ^***^*p* < 0.001.


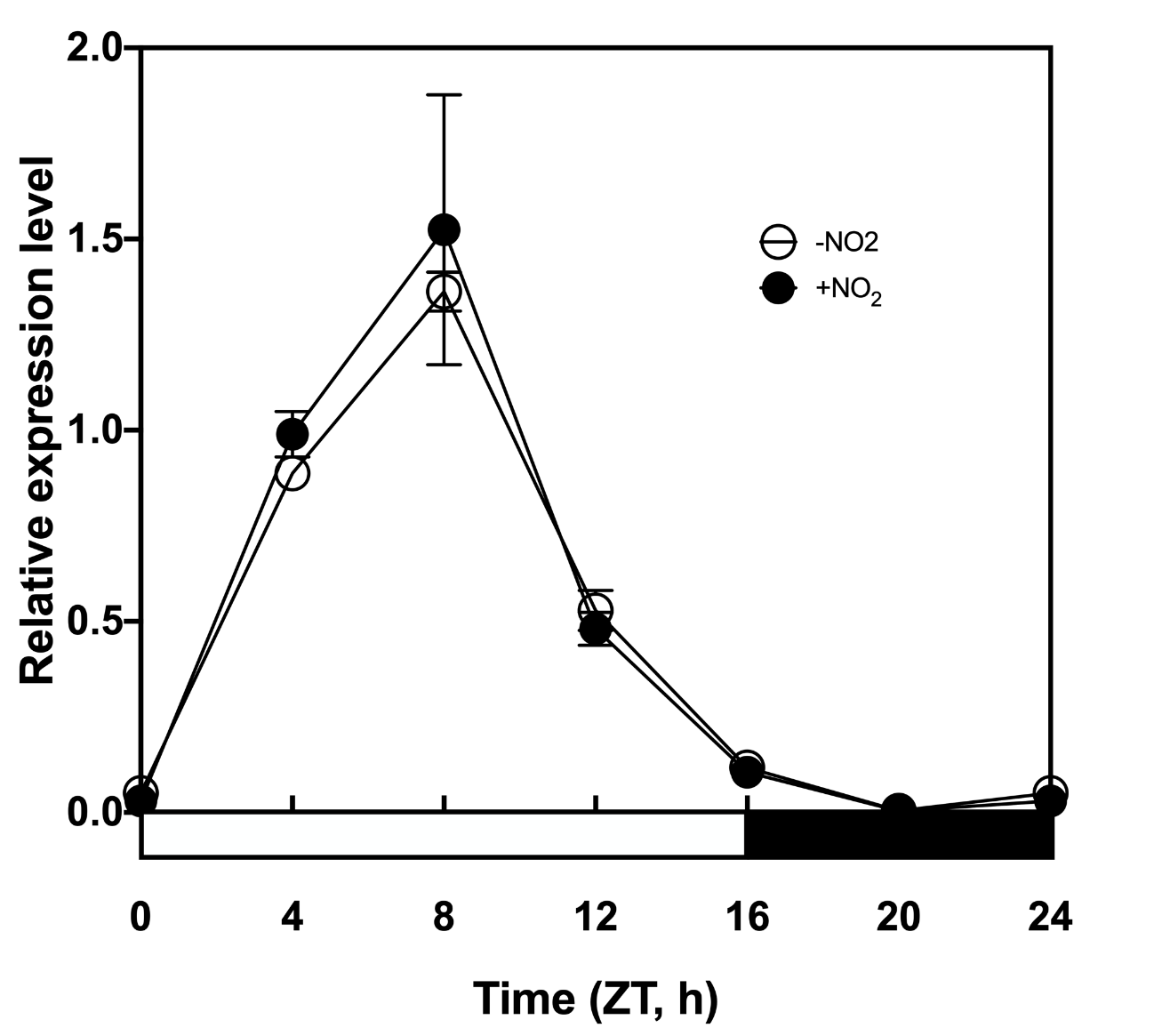


Fig. S3. Diurnal *PIF4* expression in Arabidopsis WT plants grown in the presence and absence of NO_2_ under long-day conditions (16−h light/8−h dark). Plants were grown for 8 days as described in Fig. 1. Plants were harvested and stored at −80℃ until use. The Arabidopsis *PP2A* gene was used as an internal control. Each bar represents mean ± SD of the data from four independent biological replicates.

b

a


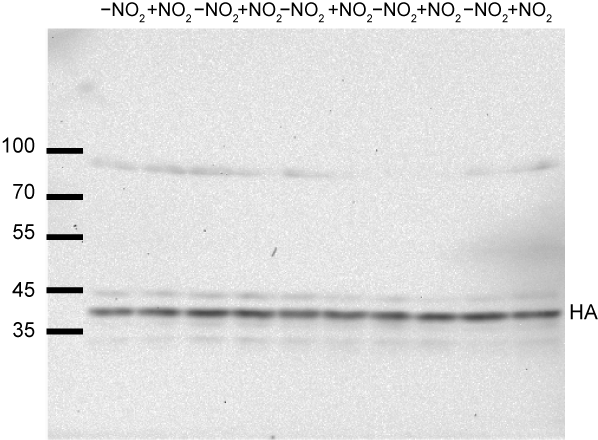

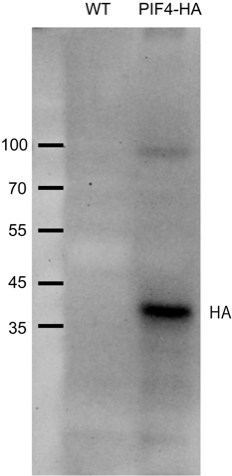


c


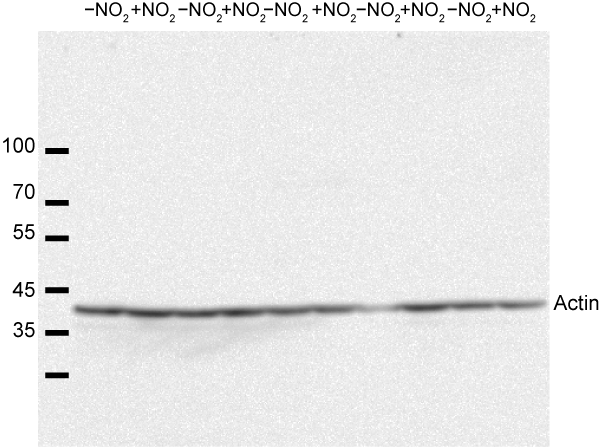


Fig. S4. Western blot analysis of PIF4-HA (a and b) and Actin (c) in Arabidopsis WT and *PIF4::PIF4-*HA plants. Plants were grown in the presence and absence of NO_2_ for 9 days as described in Fig. 1. Intensity of bands around 35 kDa in the blot with anti-HA-tag antibody (a) and intensity of bands around 40 kDa in the blot with anti-ACT11 antibody (c) were quantified. The 35 kDa band was not detected in WT plants in the blot with anti-HA antibody (b).


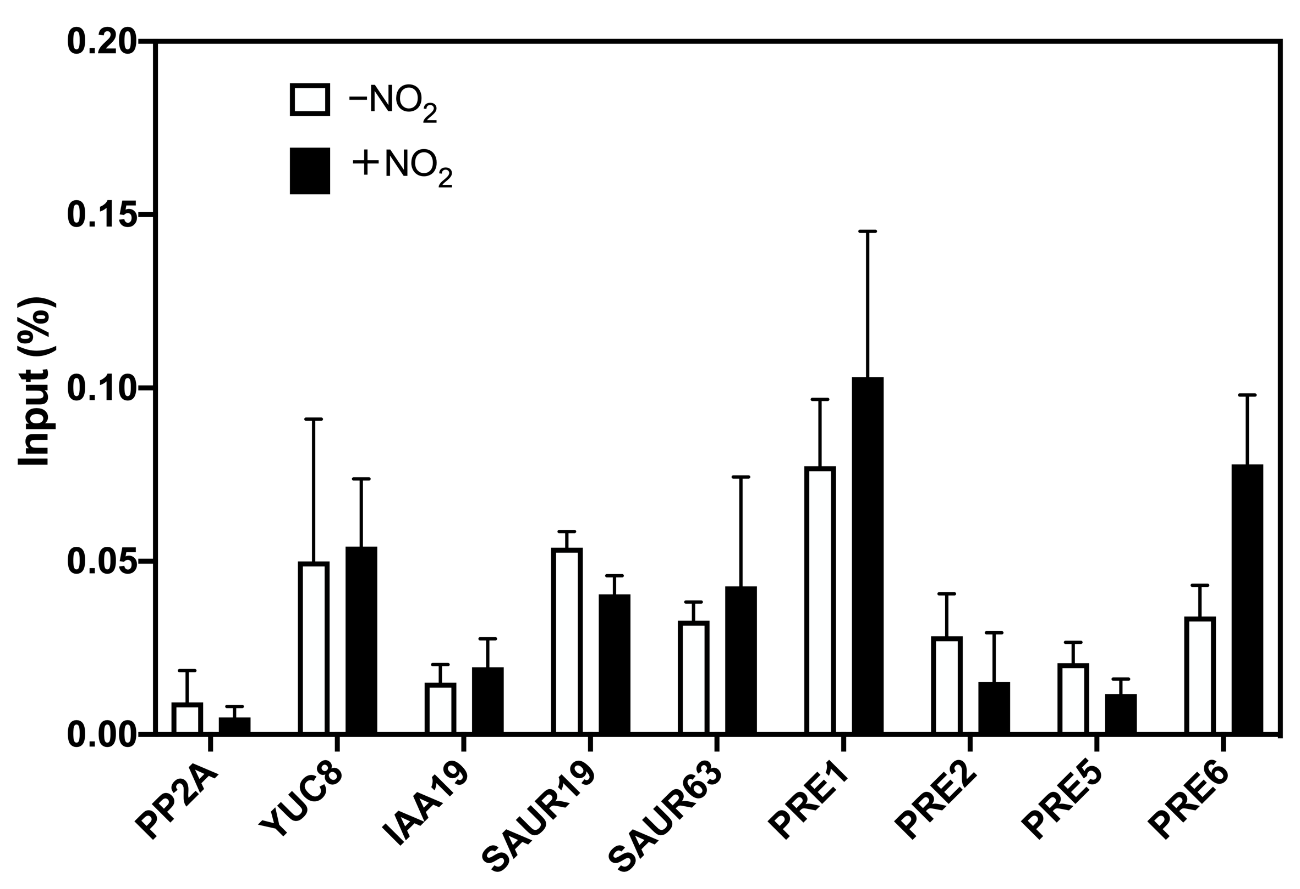


Fig. S5. Chromatin immunoprecipitation (ChIP) assays for genes other than *SAUR67* and *SAUR46* in *PIF4::PIF4*-HA Arabidopsis plants. Plants were grown in the presence and absence of NO_2_ for 9 days, as described in Fig. 1. Arabidopsis *PP2A* (load control) was used as an internal control. Plants were harvested at ZT6. Each bar represents mean ± SD of data from three independent technical replicates.

**Supporting Information**

**Table S1** List of primers used for quantitative real-time PCR and ChIP-qPCR

| Gene | Primer pairs |
| --- | --- |
| *PP2A* | 5′-TAACGTGGCCAAAATGATGC -3′ and 5′-GTTCTCCACAACCGCTTGGT -3′ |
| *YUC8* | 5′-GCCTTGCAGATAACGAGGAAAAC-3′ and 5′-CCACACAATCTCTAGTCGGTTGG-3′ |
| *IAA19* | 5′-CCAACAGTACTTGGACGAAAACAAA-3′ and 5′-TGAAGCCTTGCTTGATTCTCGG -3′ |
| *IAA29* | 5′-AGAGTCTCTGTTCCTATTGGTTGGT-3′ and 5′-CATGAAGGCAAGAACGACGAGTAA-3′ |
| *SAUR19* | 5′-ACTGCTGCTATTGGAAAGGGATTT-3′ and 5′-GGTCAAAACATCTACGGTGTGGAT-3′ |
| *SAUR46* | 5′-TCAAGCACAGGATGCTTACG-3′ and 5′-CGGACAACATCGAGGAAAGT-3′ |
| *SAUR63* | 5′-ATGAAGGTAGAGCAGGAAGTTCCA-3′ and 5′-CTGGCATGTGATAGTGACCATTCTT-3′ |
| *SAUR67* | 5′-TGGATGGAGATACAGAAAAGGCT-3′ and 5′-TGTTGAGTACTCTGTTCTTGCTGT-3′ |
| *PRE1* | 5′- AGGCAATCTTCAAGTGCTCCAAG-3′ and 5′- TACTTTCGAGGCTGATGCCTTATC-3′ |
| *PRE2* | 5′-TGATTCAGAGATTCATCCAGTGCC-3′ and 5′-TTGAGGACGTGGATACGATCTCAT-3′ |
| *PRE5* | 5′-GTCGTCGCAGAACAGTCCAAG-3′ and 5′-ACCAACAAGCACAACCATTGATTC-3′ |
| *PRE6* | 5′-TGGGAGCTTCCAGAGATGTCT-3′ and 5′-TGCTGCCCGGTTAGGACG-3′ |
| *PIF4* | 5′-TCTCCACTTCTACAGATGAACAGAG-3′ and 5′-TCTCCGATAGACCCATGTGGTG-3′ |
| *PP2A*(ChIP) | 5′-CGGCTTTCATGATTCCCTCT-3’ and 5′-GCCTTAAGCTCCGTTTCCTACTT-3′ |
| *SAUR46(ChIP)* | 5′-CTCCAATATTGACACTTCTCC-3′ and 5′-AGCTCAAGGCTCTAGCTCAGG-3′ |
| *SAUR67(ChIP)* | 5′-AACTCTTGTCATATAAACGTC-3′ and 5′-GGTTTAGACAAGCTCCTGAGC-3′ |
| *YUC8(ChIP)* | 5′-GGGAATGGGTTTGATGTGGAATT and 5′-GAGAAGGGAAGTGATGGAATTAG |
| *IAA19(ChIP)* | 5′-CGGATTCCAATGATCCAACGG and 5′-TGGGGGCAGAGACAGGTCAAC |
| *SAUR19(ChIP)* | 5′-GGGCACGTCTTATCTCAGAAACA and 5′-TGAAACTGTGGTCTTGAAGCGTG |
| *SAUR63(ChIP)* | 5′-AACTCTTGTCATATAAACGTCCA and 5′-AGGTTTAGACAAGCTCCTGAGCA |
| *PRE1(ChIP)* | 5′-GAGGGATAATGAGGGATTTCG-3′ and 5′-CTATGTCACGTGTCACCACCATGTC-3′ |
| *PRE2(ChIP)* | 5′-TGGAGATAAGAAAAGCGAGAGG and 5′-GAGCACACAAAGGTGGGACT |
| *PRE5(ChIP)* | 5′-ATTCTATGGATCCAAGGTGGGATA-3′ and 5′-TTGAGCTAACAATGTGGCCCT-3′ |
| *PRE6(ChIP)* | 5′-AGCTAAAGACACCCTTATTAAAGAAACA-3′ and 5′-GTGTTTTTATAATGCAAATTTATGAAGAGA-3′ |
